# Photon Recycling–Driven Photodetection Enhancement in Ni(AcO)_2_–MAPbBr_3_ Nanocomposite Waveguides

**DOI:** 10.1002/nap2.70196

**Published:** 2026-07-02

**Authors:** Sandra Soriano‐Díaz, Jaume Noguera‐Gómez, Diego Ramírez‐Muñoz, Pablo P. Boix, Juan P. Martínez‐Pastor, Rafael Abargues, Isaac Suárez

**Affiliations:** ^1^ Departamento de Ingeniería Electrónica Escuela Técnica Superior de Ingeniería Universidad de Valencia Valencia Spain; ^2^ UMDO Instituto de Ciencia de los Materiales Universidad de Valencia Valencia Spain; ^3^ Energy Research Institute @ NTU Nanyang Technological University Research Techno Plaza Singapore; ^4^ Instituto de Tecnología Química (Universitat Politècnica de València‐ Agencia Estatal Consejo Superior de Investigaciones Científicas) Valencia Spain

## Abstract

The reabsorption and reemision of photoluminescence (PL) photons, or photon recycled (PR) effect, has demonstrated outstanding benefits in solar cells or light‐emitting diodes. Nevertheless, this process has been unexplored in photodetectors (PD) because reliably quantifying the photocurrent driven by PL photons is technically challenging. This work demonstrates the generation of a PR‐driven photocurrent by incorporating a nickel acetate, Ni(AcO)_2_, nanocomposite containing MAPbBr_3_ (MA = methylammonium) nanocrystals (NCs) in planar waveguide structure integrating electrode contacts. The concentration of NCs in the matrix is optimized to enable efficient PL photon confinement and propagation over millimeter‐scale distances until detected. Under modulated laser illumination, this mechanism enhances photocurrent by approximately 60%. Remarkably, under nanosecond pulsed excitation, the device generates an amplified spontaneous emission signal that is reabsorbed within the film, yielding a photocurrent peak of 0.8 mA per pulse. These findings demonstrate an effective strategy for enhancing the performance of MHP‐based PDs.

## Graphical abstract

Photon recycling is demonstrated as an effective mechanism to enhance perovskite photodetector performance. A Ni(AcO)_2_/MAPbBr_3_ nanocomposite waveguide enables long‐range propagation and reabsorption of photoluminescence photons, increasing photocurrent by ∼60%. Under pulsed excitation, reabsorption of amplified spontaneous emission further enhances the photocurrent, yielding pronounced current peaks and boosting device response.

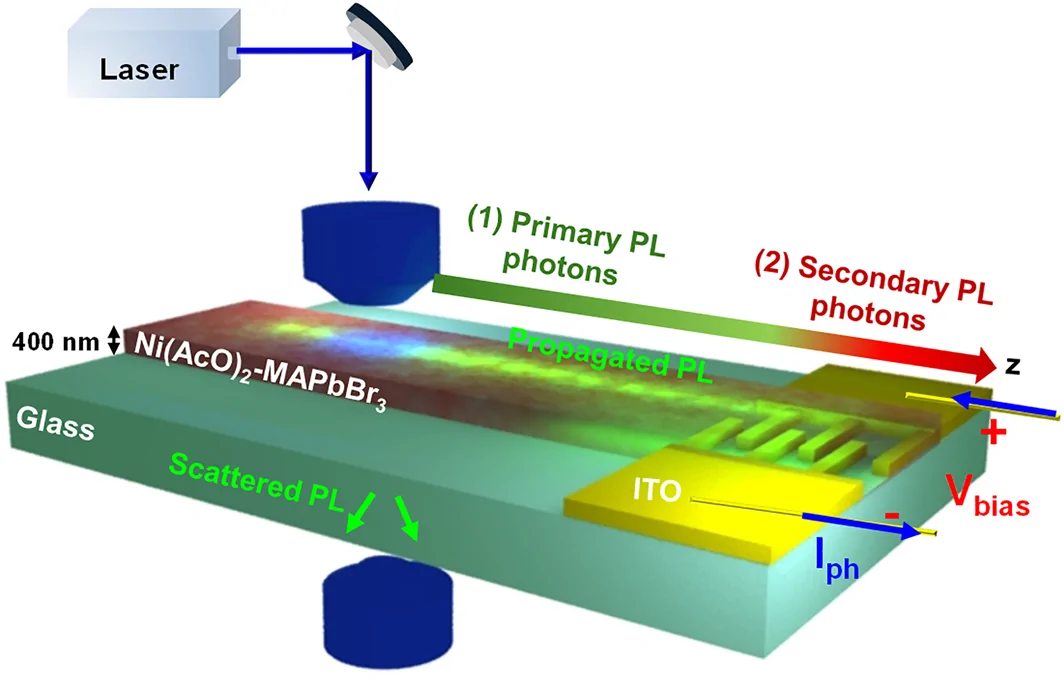

## 1 Introduction

Photon recycling (PR) is a process in which photons emitted by a semiconductor experience several (re)absorption and (re)emission cycles before escaping the material [1, 2]. This mechanism increases the radiative lifetime and diffusion length of photogenerated carriers [3], thereby impacting the performance of optoelectronic devices [4]. PR is particularly effective in metal halide perovskite (MHP) due to their characteristic high quantum yield (QY) of emission, strong absorption above the bandgap edge, and remarkable overlap between the absorption and emission spectra [5]. Demonstrated benefits include enhanced photoluminescence (PL) emission [6], increased open voltage and power conversion efficiency of solar cells [7, 8, 9, 10] as well as improved electroluminescence quantum efficiency of light‐emitting diodes [11, 12].

In this scenario, PR is also expected to play a key role in improving the performance of MHP photodetectors (PDs), which already combine a low‐cost and straightforward fabrication with excellent figures of merit, such as a high responsivity, spectral tunability, high linear dynamic range, fast response time, or low noise current [13, 14, 15, 16, 17]. Therefore, evaluating the contribution of the PR to the photocurrent (*I*_ph_) generated after light excitation, which is essential to further optimize MHPs PDs. To date, reabsorption of emitted photons has been shown to enhance the *I*_ph_ and responsivity in CdS/CdSSe nanowire PDs, involving semiconductor nanostructures with different bandgap [18]. By analogy, similar effects are anticipated in MHP‐based devices where PR can arise from the intrinsic inhomogeneous broadening of nanocrystal (NC) ensembles, enabling preferential reabsorption of photons emitted by smaller NCs with slightly larger bandgaps [19]. However, to the best of our knowledge, this phenomenon has not been reported for MHPs and remains elusive to other semiconductors. Indeed, the quantification of the *I*_ph_ driven exclusively by recycled photons is challenging as the photodetected signal is typically dominated by contributions from directly photogenerated carriers, whereas the PL‐induced current is typically below the detection limit of standard measurement methods. For MHP PDs, a typical responsivity reported above the bandgap can be higher than 1 mA/W [20]. Then, for an incident pump beam of 10 mW, this responsivity corresponds to I_ph_ ∼10 μA, which can be measured using standard I‐V characterization methods. However, at the bandgap edge, where the PL peak is located, the responsivity drops significantly, often to 10–100 μA/W, yielding only *I*_ph_ = 0.1–1 μA for the same device and excitation conditions. Moreover, the PL photons contributing to the charge generation are significantly reduced by several loss mechanisms. For example, the nonradiative recombination in the MHP films usually limits the PL quantum yield (PLQY) to 10%–50% [21, 22], the parasitic absorption introduced by the electrodes can attenuate another 10%–20% of the PL signal, [11, 12], and another 10%–20% of the photons will be lost by the critical angle [11, 12]. More importantly, most of the PL photons will be diffused along the MHP film outside the electrode gap; that is considering in‐plane omnidirectional emission, only 3% of the photons generated on 1 mm^2^ spot will be directed in an electrode gap of 100 μm overlapping the spot. Under these conditions, the I_ph_ generated by reabsorbed PL photons is expected to be extremely low, *I*_ph_ < 1 nA, which demands more sensitive instrumentation and a careful suppression of the dark and noise currents.

In this work, we propose an elegant strategy to increase the *I*_ph_ level generated by reabsorbed PL photons based on the generation of amplified spontaneous emission (ASE) in a waveguide configuration. Typically, a waveguide scheme represents a favorable platform to propagate flux PR over low distances, ∼ 1 mm, as the confinement and directionality of guided modes facilitate the propagation of photons and increase the light–matter interaction [19, 23, 24, 25, 26]. Under these conditions, integrating a PD into a waveguide structure offers an interesting approach to redirect a high‐intensity flux of PL photons toward the electrode gap. Moreover, the waveguide configuration also provides an optimal platform to overcome the ASE threshold, which, combined with PR, is expected to substantially enhance the population of low‐energy PL photons [27, 28] that potentially can increase the level of *I*_ph_. In this context, we have recently demonstrated a planar waveguide structure where the ASE threshold is significantly reduced by the flux of recycled PL photons [28]. The device was based on a nickel acetate matrix (Ni(AcO)_2_) containing MAPbBr_3_ (MA: methylammonium) MHPs NCs [29]. This multifunctional nanocomposite is synthesized under a low‐cost and straightforward route where the MAPbBr_3_ NCs are in situ crystallized inside Ni(AcO)_2_ during the spin‐coating deposition, resulting in thin films with PLQY of 80%, with excellent ambient and optical stability. Moreover, the optical properties can be easily tuned by varying the concentration of MHP within the Ni(AcO)_2_ matrix [29, 30, 31].

In the present work, the MAPbBr_3_‐Ni(AcO)_2_ waveguide is integrated with interdigital electrodes to study the *I*_ph_ generated via PR. For this purpose, an optimal filling factor (ff) of MAPbBr_3_ NCs in the matrix (ff = 0.4) is chosen to ensure high PLQY, ASE generation, and the propagation of recycled photons over long distances (1–6 mm) based in our previous studies [28]. This concentration leads to low dark currents (< 1 nA at 1 V) allowing for the detection of low *I*_ph_ values. Under these conditions, PR results in a crucial mechanism to reduce optical losses and amplify photoconversion, enabling higher sensitivity and a boost of detected light by more than 60%, when the device is pumped with a modulated laser (1 kHz, duty cycle 50%, i.e. periodical modulation). Furthermore, under upon optical pumping at the nanosecond scale and sufficiently high fluences, the flux of recycled photons undergoes ASE generation, which further boosts *I*_ph_ to the range of ∼1 mA/pulse. This strategy can be used to enhance the performance of MHP PDs and paves the road for special applications, such as color or multispectral imaging technologies.

## 2 Device Design

The waveguide‐PD used in this work consists of a 400 nm‐thick MAPbBr_3_‐Ni(AcO)_2_ coated on patterned ITO electrodes, see the details of the fabrication in the experimental methods. Each substrate contains five sets of three interdigitated electrodes with a fixed gap of 50 μm connected to 2 × 2 mm^2^ paths, see Figure 1a. The nanocomposite exhibited excellent operational and environmental stability, with no appreciable degradation in electrical or optical performance observed over extended storage times [29] and during repeated measurements, see Supporting Information S1: Figure S1 and S2. A bias voltage (*V*_bias_) applied to the paths promotes the collection of a given *I*_ph,_ whose level depends on the illumination in the interdigital electrodes, as shown in Figure 1b. To properly characterize the *I*_ph_ generated by PR (i.e., to isolate the contribution of PR in the measured *I*_ph_), the nanocomposite must simultaneously ensure efficient PL emission and propagation as well as sufficient conductivity for *I*_ph_ extraction. Regarding emission efficiency, we recently demonstrated that an optimal ff range of ff = 0.07–0.4 of MAPbBr_3_ in the Ni(AcO)_2_ matrix results in a PLQY of 80% [31], and more importantly, it enables ASE under illumination of ns pulses of ∼1 μJ/cm^2^ in a waveguide geometry [28]. Outside this range of concentrations, the emission efficiency drops significantly due to either a low concentration of emission centers (ff < 0.07) or the formation of trap states introducing nonradiative channels (ff > 0.4) [28]. Since the Ni(AcO)_2_ matrix is inherently non‐conducting, a percolation threshold concentration of NCs is expected to sustain an appropriate carrier transport for photodetection [32, 33]. Then, within the aforementioned range of concentrations, ff = 0.4 is chosen as an optimal composition to ensure both strong PL and an appreciable effective carrier mobility (see next section). According to our previous results [28], the refractive index of the nanocomposite with this concentration (ff = 0.4) reaches 1.82 at the maximum PL wavelength. The resulting refractive index contrast between the nanocomposite and the surroundings (n = 1.52 for glass and *n* = 1 for air) is high enough to conform a planar waveguide at both transverse electric (TE) and transverse magnetic (TM) modes. See Supporting Information S1: Table S2 and the analysis with a transfer matrix algorithm [34] in the Supporting Information S2. The waveguide geometry favors TE‐polarized fundamental modes, whereas higher‐order modes are close (or below) cut‐off and exhibit insufficient confinement to be efficiently excited under the experimental conditions [28]. Including a 100 nm ITO electrode layer in the model yields similar mode profiles and propagation losses, demonstrating that ITO has a negligible impact on light propagation. Besides, the confinement factor, defined as the ratio between power distribution within the active region (i.e., nanocomposite) versus the total power distribution, reaches a value ≈ 85%, indicating that most of the PL photons will travel in the active medium, as illustrated by the TE_0_ mode distribution in Figure 1c. This strong confinement favors directional light propagation and enhances absorption and emission efficiencies, ultimately reinforcing the PR effect [19].

**FIGURE 1:**
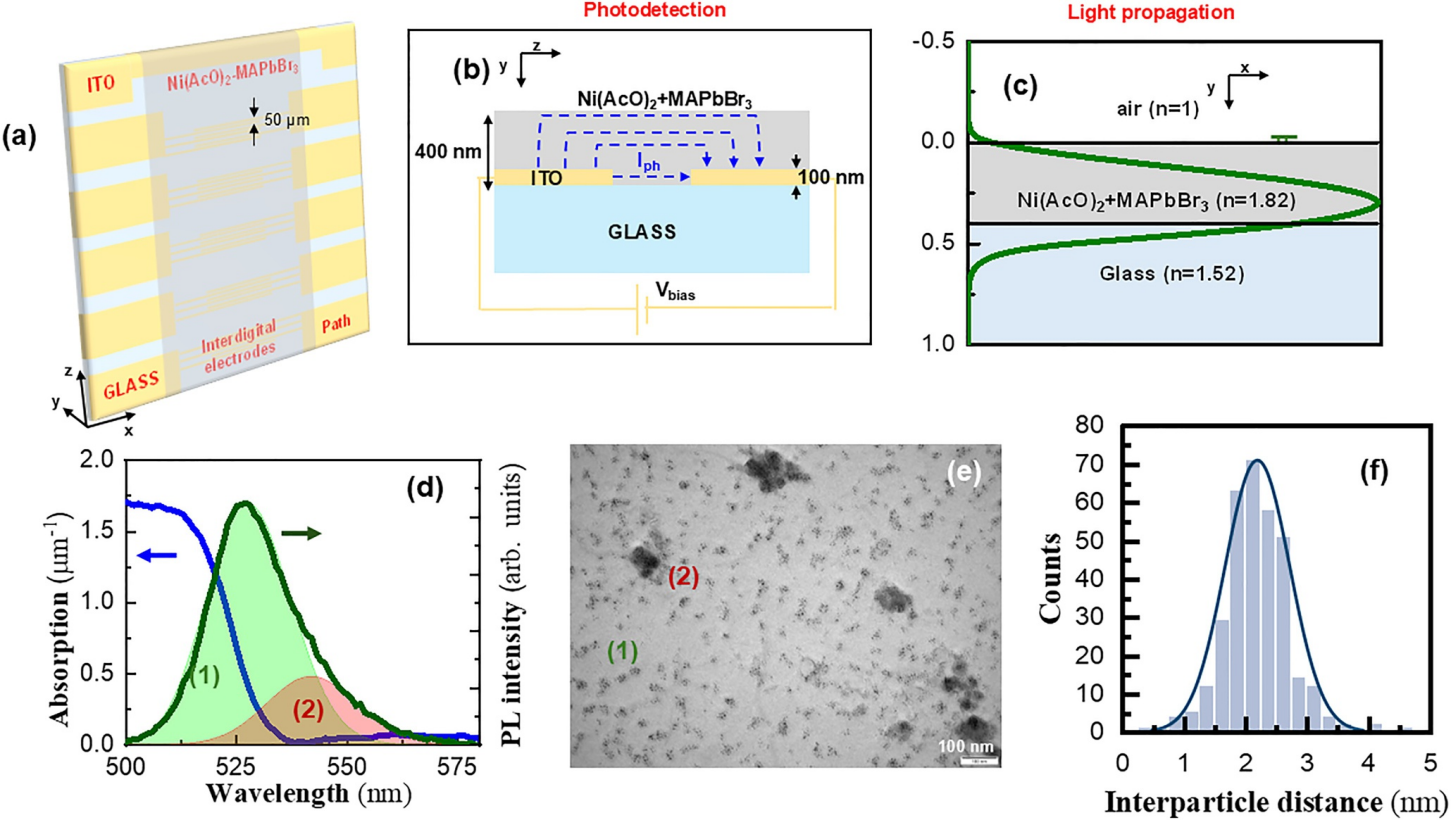
(a) Scheme of the PD device. (b) Cross section. (c) Power distribution of the TE_0_ mode. (d) Absorption (blue and left axis) and PL (green and right axis) spectra of the nanocomposite. PL is deconvoluted by two Gaussian distributions, labeled (1) and (2). (e) TEM image of the Ni(AcO)_2_:MAPbBr_3_ nanocomposite composed by big, (1), and small, (2), NCs. (f) Statistics of the interparticle distance between NCs in each cluster.

The absorption spectrum of the nanocomposite with ff = 0.4 exhibits a band edge at 510 nm and an absorption coefficient (*α*) of *α* = 1.75 μm^−1^ above the bandgap, as shown by the blue line in Figure 1d. The PL spectrum, green line in Figure 1d, can be deconvoluted in two Gaussian distributions centered at 530 nm (B1) and 545 nm (B2) overlapping the absorption spectra at *α* = 0.25 μm^−1^ and *α* = 0.0485 μm^−1^, respectively. Importantly, Figure 1e shows a Transmission Electron Microscopy (TEM) image of the nanocomposite thin film, revealing two different NC populations with average diameters of 5 and 25 nm, see Supporting Information S1: Figure S3, which are associated with the B1 and B2 emission bands, respectively [28]. Then, the significant reabsorption of the light emitted by the small NCs will result in a significant flux of recycled photons, which can eventually increase the B2 signal [28]. Indeed, the short propagation length, *L*_P_ = 1/*α*, of the B1 band *L*_P_ = 4 μm, suggests that for standard propagation distances in a waveguide, 1–6 mm, PL photons can undergo multiple reabsorption–reemission cycles, enabling an observable PR effect. The photodetection of this flux of PR photons critically depends on the charge transport between the NCs throughout the Ni(AcO)_2_ matrix. For this purpose, TEM images reveal two characteristic length scales in the nanocomposite: short interparticle separations (∼1 nm) within local NC clusters and larger separations (∼50 nm) between neighboring clusters. Within these clusters, see zoomed image in Supporting Information S1: Figure S4, the short NC–NC separations of approximately 1–3.5 nm (see Figure 1f) govern the local hopping distances for carrier transport [35, 36]. In contrast, the larger separations between clusters suggest a heterogeneous morphology in which direct electronic transport through the insulating matrix is hindered. At these conditions, long‐range electrical connectivity is likely established through sparse percolation pathways that are not fully resolved by TEM, where the shortest interparticle distances preferentially define the conductive paths. In this way, PR and optical re‐excitation processes are expected to play a crucial role in enabling long‐range transport across the nanocomposite by regenerating charge carriers in spatially separated conductive regions, thereby contributing to the measurable *I*_ph_.

## 3 Characterization of the Photodetection Properties

To understand the carrier transport in the nanocomposite under study, it is essential to investigate its electrical properties and its photodetection performance under external illumination. First, the complex impedance of the nanocomposite PD, Z (j*ω*), was measured in dark conditions using the set‐up illustrated in Figure 2a. A signal generator is connected in series to one of the contact paths, whereas the other contact is bound to a shunt resistance (100 k*Ω*) to close the circuit. Then, Z (j*ω*) is determined using the relation [*v*_in_ (j*ω*) − *v*_out_ (j*ω*)]/*i* (j*ω*) by sweeping the signal generator frequency (*f*) at a fixed amplitude and measuring the voltage across the shunt resistance with a lock‐in amplifier synchronized with the signal generator. For a frequency range between 10 Hz and 3 MHz, the nanocomposite exhibits a low‐pass filter response. The impedance remains nearly constant at |Z (jω)| = 160 dBΩ for *f* = 50–1000 Hz and presents a cut‐off frequency at f_−3dB_ ≈ 10 kHz, as observed in Figure 2b. Beyond this point, |Z (jω)| decreases with a slope close to 20 dBΩ/decade until *f* ≈ 1 MHz where |Z (jω)| saturates at |Z (jω)| = 120 dBΩ. This behavior can be nicely fitted (continuous line in Figure 2b) using the equivalent circuit shown in the inset of Figure 2b with parameters *R*_0_ = 0.9 MΩ, *R*_1_ = 115 MΩ, and *C*_1_ = 1.4 nF. The phase of Z (jω) exhibits the complementary low‐pass filter shape, and it is also fitted with the same parameters with only minor deviations at high frequencies, see Figure 2c. A similar response is observed for a nanocomposite with ff = 0.2 but with larger resistance values (*R*_0_ = 1.1 MΩ, *R*_1_ = 900 MΩ and *C*_1_ = 1.1 nF, see Supporting Information S1: Figure S5), indicating a lower conductivity by decreasing ff, as indicated above. In both cases, fractional capacitors with impedance *Z*_c_ = (1/jωC)^0.9^ were used to improve the fitting [20].

**FIGURE 2:**
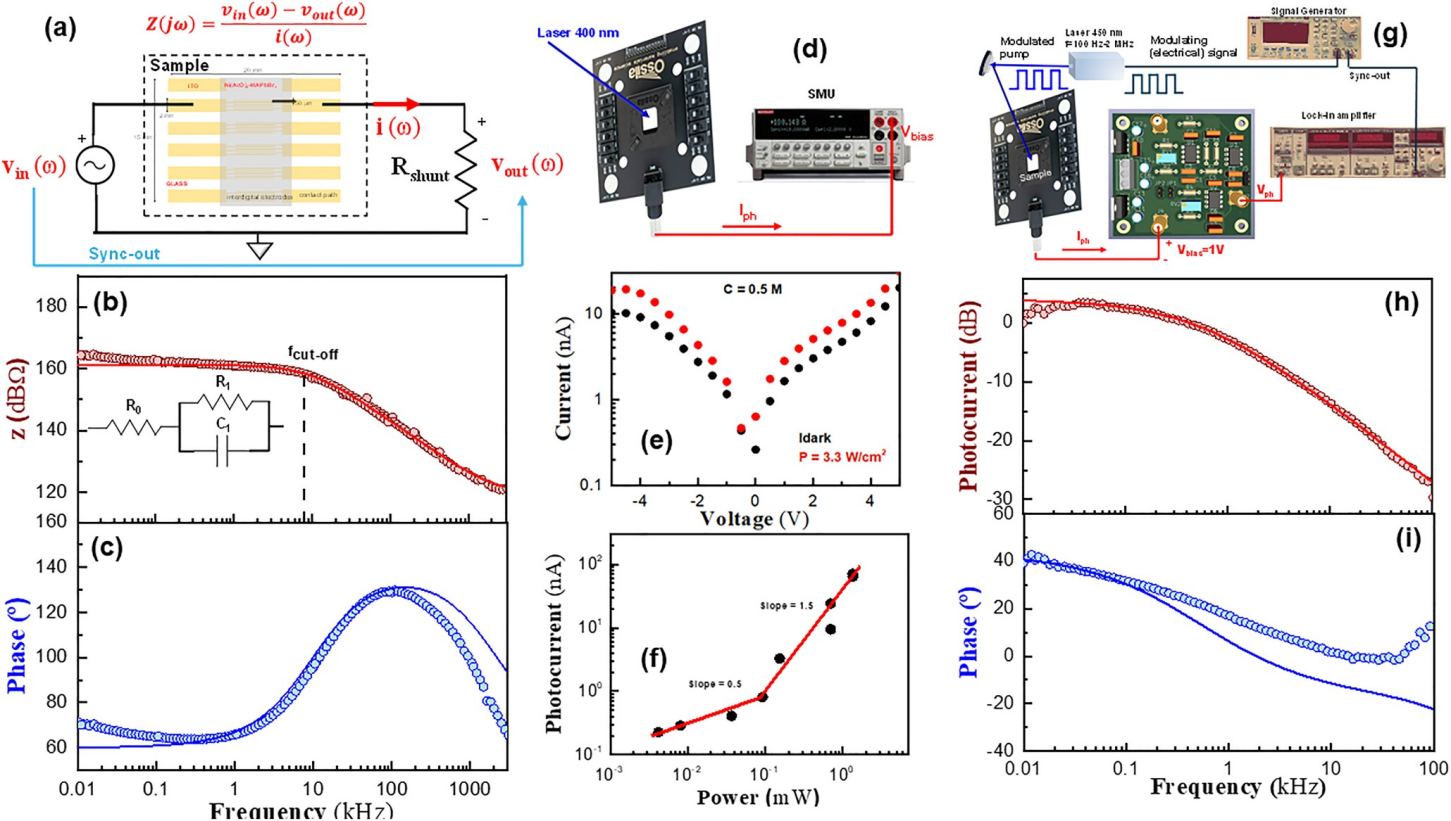
(a–c) Characterization of the impedance (Z (jω)) of the nanocomposite deposited on interdigital electrodes. (a) Set‐up. (b) |Z (jw)| (c) phase of Z (jw). (d–f) Characterization of *I*_ph_ under CW excitation. (d) Set‐up. (e) I–V curve under dark (black) and illumination (red) conditions. (f) *I*_ph_ as a function of the excitation fluence. (g–i) Characterization of the frequency response of *I*_ph_. (g) Set‐up (h) Amplitude. (i) Phase. Brown and blue colors correspond to the magnitude and phase of *I*_ph_, respectively.

To investigate phototransport properties, the interdigitated photodevices based on the nanocomposites with ff = 0.4 were examined using an electric test board connected to a source and measure unit (SMU) under dark and illumination conditions, as illustrated in Figure 2d. In dark conditions (black symbols in Figure 2e), the PD exhibits a dark current of 1–10 nA across a voltage range of 1–4 V. The log‐log plot in Figure 2e reveals that the PD device behaves as a linear resistance for the [−2 V, 2 V] range. Thus, to ensure that the device works in the ohmic linear regime, a bias of *V*_bias_ = 1 V is fixed in experiments under illumination. A dark resistance of 375 MΩ can be deduced from the slope in the [−2 V, 2 V] linear regime, which is a value of the same order of magnitude to that obtained in the impedance characterization shown in Figure 2b–c. Considering the electrode gap of 50 μm and a film thickness of 400 nm, this resistance corresponds to a resistivity of around 2300 Ω cm, which is not far from that reported for MAPbI_3_ polycristalline thin films [20]. Samples with smaller concentration, ff = 0.2, demonstrated lower dark current, *I*_dark_ < 2 nA for the 1–4 V voltage range, see Supporting Information S1: Figure S6, again indicating a higher resistivity (≈ 4600 Ω cm). As expected, the NC concentration in the matrix can tune the effective resistivity of the material playing an important role in the PD capabilities. Indeed, the films under continuous wave (CW) illumination at 404 nm required a minimum concentration, ff > 0.2, to obtain a detectable *I*_ph_. For ff = 0.4, at *V*_bias_ = 1 V and an excitation fluence *P* = 3.3 W/cm^2^, *I*_ph_ ≈ 2 nA, resulting on *I*_ph_−*I*_dark_ ≈ 1 nA and an ON‐OFF ratio of *I*_ph_/*I*_dark_ = 2. These values corresponds to a responsivity of ≈ 20 nA/W and a detectivity of ≈ 2·10^5^ Jones. Although these figures of merit lower than those typically reported for conventional MHPs photodetectors [20], the remain sufficiently high considering that they are achieved in a nanocomposite system where the dielectric matrix inherently limits electron transport. Indeed, for ff = 0.2 a limited photoresponse is observed, that is *I*_ph_−*I*_dark_ < 0.1, see Supporting Information S1: Figure S6. Here, since charge carriers must traverse the poorly conducting Ni(AcO)_2_ to move between neighboring NCs, electron hopping is proposed as the dominant transport mechanism rather than bandgap transport or carrier diffusion that are typically chosen to explain conductivity in polycristalline thin films [37, 38]. As in any multicomponent material of a semiconductor dispersed in an insulating matrix, the overall electrical conductivity follows the percolation theory, where charge transport occurs only when the concentration of conductive domains exceeds a critical threshold (i.e., the percolation threshold), forming continuous paths across the material [39, 40]. A similar interpretation has been reported for closed packed films of PbS [35, 36, 41]. Supporting Information S1: Figure S7 shows the typical ON‐OFF characterization for different excitations, showing a progressive increase of *I*_dark_ after illumination from extremely low values (*I*_dark_) to 2 nA for the highest intensities and measurement time. The shape of the ON‐OFF also suggests a low decay component (∼1 s), which takes *I*_ph_ before stabilizing on the plateau that determines the performance of the device. This behavior can be understood by the self‐trapping‐detrapping mechanism of electron and holes characteristics of perovskite NCs [42]. Nevertheless, this effect is neglected in the results presented above because all measurements were integrated sufficient time to ensure stabilization. The *I*_ph_−*P* curve of the device deduced from the ON‐OFF characterization, Figure 2f, shows a sublinear dependence (*I*_ph_ ∝ *P* ^0.5^) below 0.1 mW and a super‐linear dependence (*I*_ph_ ∝ *P* ^1.5^) in the 0.1–3 mW range. Here, we believe that ∼0.1 mW corresponds to the percolation threshold at which the density of photogenerated carriers becomes sufficient to establish efficient transport pathways between NCs [43]. Above this threshold, the material exhibits appreciable conductivity and I_ph_ follows a quasi‐linear dependence with P, indicating a low carrier injection regime.

Finally, the frequency response of the *I*_ph_ was measured with a transimpedance amplifier connected with a lock‐in amplifier synchronized with the excitation, see scheme in Figure 2g and the details of the experiment in reference [20]. Excitation fluence was fixed to 3.3 W/cm^2^ to ensure that the device is above the percolation threshold and the integration time was chosen to be sufficiently long enough to allow the trapping/detrapping process to reach a steady state regime. The amplitude and phase of *I*_ph,_ indicate a low‐pass filter behavior with a cut‐off frequency of 0.33 kHz, as shown in Figure 2h–i (data symbols), indicating a transient time of 3.3 ms. In the 10 Hz‐100 kHz range, experimental data can be nicely fitted, solid lines in Figure 2h–2i, by the transfer function composed of three fractional poles, associated with the cut‐off frequency, the intrinsic capacity of the device, and the RC constant of the conditioning circuit [20], Supporting Information S6 contains the transfer function and all fitting parameters. For *f* > 100 kHz, the signal was too weak to obtain reliable data.

## 4 Photodetection of Recycled Photons

To analyze the *I*_ph_ generated by PR photons, the device must efficiently excite PL and sustain its propagation from the excitation point and the contact electrodes [44]. A waveguide is a suitable geometry for this purpose, as it enables PL to propagate over several millimeters, allowing multiple absorption–emission cycles and thereby producing a significant flux of recycled photons [19, 23, 24, 25, 26]. Besides, a waveguide geometry also benefits the photodetection of the wavelengths at the absorption edge (or bandgap energy) [45, 46], although this approach has been unexplored with PL emitted photons by the nanocomposite, as carried out in the present case. In this context, the air/Ni(AcO)_2_:MAPbBr_3_/glass structure proposed in this work has demonstrated excellent characteristics for PR, suggesting to be an adequate architecture to establish a current from recycled photons [28]. To investigate the spatially resolved photocurrent and photoluminescence response of this structure, the excitation beam was focused (excitation spot ∼100 μm^2^) onto the sample surface using a microscope objective mounted on a XYZ translation stage. This configuration allows a shift of the excitation position along the waveguide axis (*z*) from regions outside the electrodes to the electrode area (Figure 3a). For each excitation position, *z*_0_, *I*_ph_ was measured as a function of both excitation fluence and spot position using a source‐measure unit (SMU) under a constant applied bias of *V*_bias_ = 1 V. To obtain information of the PL signal, a second microscope objective is positioned below the glass substrate to collect the forward‐scattered PL.

**FIGURE 3:**
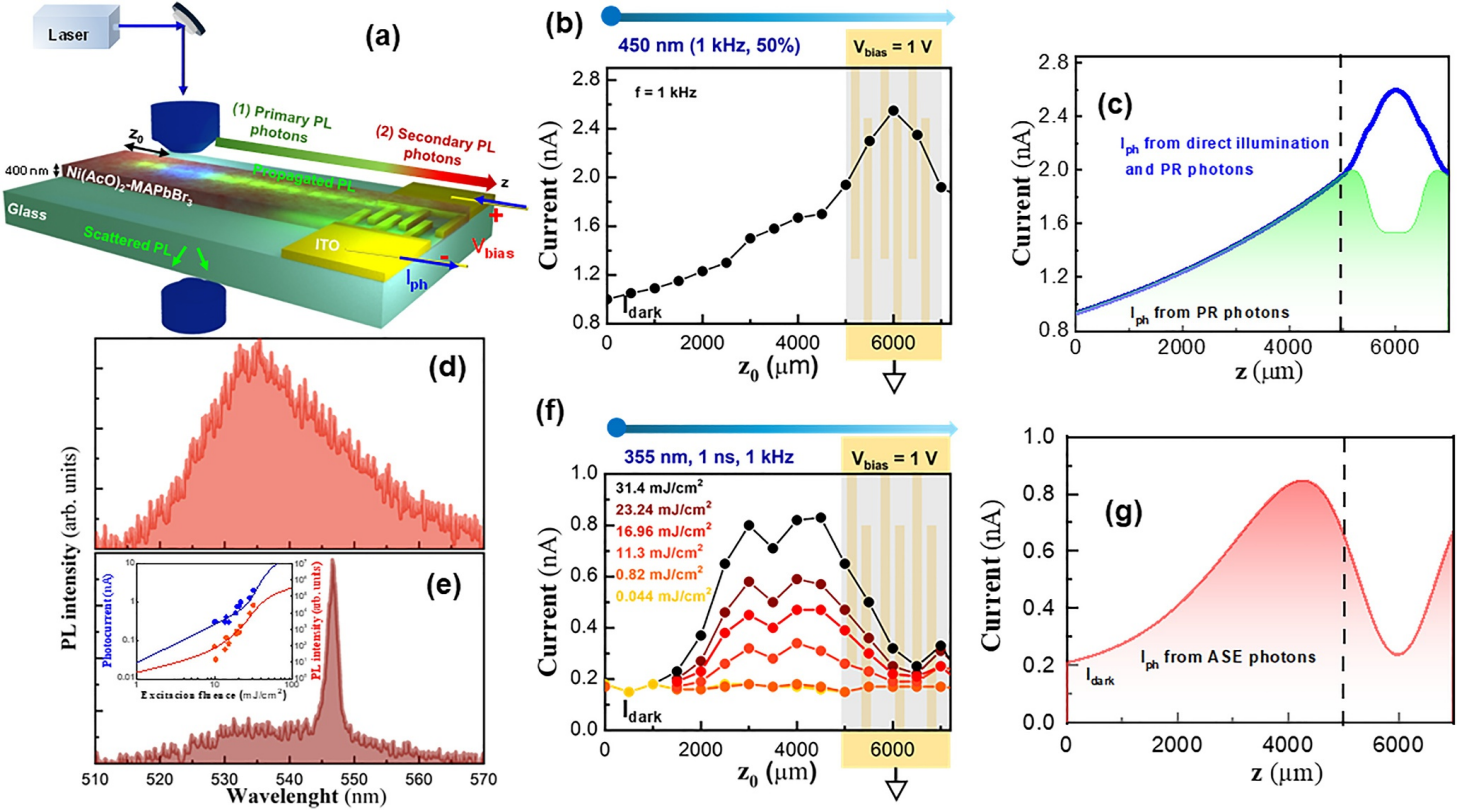
*I*_ph_ as a function of the distance between the spot and the contacts (z). (a) Experimental set‐up. (b) *I*_ph_ obtained with a modulated laser at 450 nm 1 kHz (black symbols) and 100 Hz (red symbols). (c) Modeling of the data illustrated in Figure 1b. PL spectra measured by forward scattering geometry (d) P < *P*th. (e) P > *P*th (f) I_ph_ obtained with a pulsed laser 355 nm (1 ns, 1 kHz). (g) Modeling of the data illustrated in Figure 1d.

At these conditions, the dependence of *I*_ph_ with *z*_0_ is modeled by solving coupled carrier rate‐photon propagation equations under spatially resolved optical excitation, see Supporting Information S7. The simulation considers local excitation of the device surface by a focused pump beam, whose position, *z*_0_, is scanned along the waveguide. For each *z*_0_, the model calculates, along the waveguide axis, the generation and propagation of two families of PL photons, B1 at 530 nm and B2 at 545 nm. For this purpose, each photon family is characterized by its corresponding effective reabsorption coefficient, and for stimulated emission conditions, by a gain coefficient in B2. Then, at each propagation step, carriers are generated through different contributions: (i) direct excitation from the pump beam, (ii) reabsorption of B1 photons associated with photon recycling, and (iii) reabsorption of B2 photons associated with ASE. Since charge transport in the nanocomposite is limited by hopping between NCs, each contribution acts as an effective transport channel with a different spatial redistribution efficiency. Consequently, the measured *I*_ph_ results from the interplay between carrier lifetime, hopping‐limited transport, and photon‐mediated excitation redistribution along the waveguide, which together define an effective carrier density that accounts separately for the contribution of each channel. In this way, *I*_ph_ is calculated by integrating the effective carrier density over the electrode region.

The first experiment was conducted using a periodically modulated laser with a frequency of 100Hz‐1 kHz (duty cycle 50%) at an excitation power density of *P* = 3.3 W/cm^2^. The corresponding density of electron hole pairs (*N*_eh_) is estimated as *N*_eh_ = *P*·*τ*·*α*/hν, where *τ* is the lifetime, *α* and *hν* the energy of the photon. Considering *τ* ≈ 1 ns and *α* ≈ 2 μm^−1^ [28], the estimated carrier density is *N*_eh_ ≈ 5.2·10^13^ cm^−3^, which is well below than the ASE threshold reported for MHPs in waveguides (> 10^16^ cm^−3^) [47]. At *f* = 1 kHz, *I*_ph_ yielded a maximum when the laser beam illuminated the electrode region. Conversely, a quasi‐exponential tail was observed when the beam was shifted away from the electrodes, as depicted in Figure 3b. Similar curves were obtained for *f* = 100 Hz, see Supporting Information S1: Figure S8.

Given that this excitation regime is below the ASE threshold, direct local excitation and photon recycling are the two channels that can generate the measured *I*_ph_. In a conventional PD system, direct local excitation is expected to produce *I*_ph_ when the spot illuminates the contacts area, *z* = 5000–7000 μm in Figure 3b. Nevertheless, when the excitation occurs outside the electrode region, *z* = 0–5000 μm, direct absorption can only contribute through an exponential term governed by the carrier diffusion length, which in perovskite materials is reported to be only ≈ 1 μm [37, 38]. Then, the observation of remarkable *I*_ph_ over millimeter‐scale distances respect the electrode region indicates that a second mechanism contributed in the measured *I*_ph_.

Outside the contact area, *I*_ph_ can be fitted with an exponential dependence *I*_ph_ can ∝ exp (−*z*/*z*_0_) with *z*_0_ = 5.6 mm. This characteristic length can be interpreted as an effective transport length associated with recycled photons propagating along the waveguide [19]. In this way, PR enables excitation transport over remarkably long distances, on the order of millimeters [19], and constitutes the dominant mechanism for the *I*_ph_ generated outside the electrode area.

The coupled carrier‐photon transport model accurately reproduces the experimental data assuming an effective absorption coefficient of *α*_1_ = 10 cm^−1^ and considering that only 20% of carriers generated under direct excitation contribute to the measured *I*_ph_. In the nanocomposite under study, charge transport is limited by hopping between NCs. This effect mainly affects the channels induced by direct excitation where the fast recombination time (1 ns), typically similar or faster than the hopping time [48, 49], provokes the losses of carriers by radiative or nonradiative recombination. In contrast, repeated photon recycling events across the entire B_1_ emission band generate a substantial flux of recycled photons that continuously regenerates carriers along the structure (blue area in Figure 3c) and which cumulative effect is modeled with the effective absorption coefficient of *α*_1_ = 10 cm^−1^. As a result, the simulation shows that recycled photons enhance *I*_ph_ by 60% when the spot illuminates the electrode area and nearly 100% when the spot is shifted away. The model also reveals an electron mobility and intrinsic carrier density of μ = 7.8·10^−4^ cm^2^·V^−1^·s^−1^ and *n*_0_ = 5.4·10^11^ cm^−3^, respectively. The relatively low values of μ deduced here compared to the mobilities reported for perovskite thin films of monocrystals (1–100 cm^2^·V^−1^·s^−1^) [38, 50] are reasonable considering the low conductivity of the nanocomposite. Finally, the simulation carried out considering traps indicate that only high trap densities influence the generated I_ph_, see Supporting Information S1: Figure S9.

When the sample is illuminated with a 1 ns pulsed laser at the same repetition frequency (1 kHz) and sufficient excitation energy (P), the nanocomposite exhibits a collapse of the spontaneously emitted PL (Figure 3d) into a narrow (FWHM ≈ 10 nm) ASE band at 545 nm, see Figure 3e. According to our previous results [28], this band originates from the amplification of photons emitted by the bigger NCs (B2 band), which is further enhanced by the reabsorption of the photons emitted by the small NCs (B1 band). Indeed, the ASE threshold (*P*th) takes place at around 10 mJ/cm^2^, consistent with previous forward scattering measurements [28]. In the present work, the ASE is exploited as an efficient mechanism to enhance *I*_ph_ by the optical amplification of recycled photons. In this experiment, the ASE threshold marks the onset from which *I*_ph_ is detected (see inset in Figure 3e). More interestingly, for all excitation fluences above the threshold, the maximum I_ph_ occurs when the illumination spot is shifted Δz ≈ 2–2.5 mm from the electrode area, as shown in Figure 3f. In particular, the higher intensity of the ASE band boosts the *I*_ph_ up to 0.8 nA, which normalized by the duty cycle of the pulsed laser (10^−6^) corresponds to a remarkable peak current of *I*_ph_ = 0.8 mA/pulse. At the same time, the *I*_ph_ difference between that measured at the maximum (*z* ≈ 4 mm) and the electrodes (*z* ≈ 6 mm) increases linearly with *P* above P > *P*th, as illustrated in Supporting Information S1: Figure S10.

These results indicate that, among the different mechanisms responsible of charge transport (direct illumination, photon recycling and ASE), ASE dominates the generation of *I*_p_. Again, to measure an appreciable *I*_ph_ the three charge transport channels compete with the hopping time between NCs, reported to be ∼1 ns for colloidal QDs [48, 49]. Under direct pulsed illumination, the short pulse duration (1 ns) together with the fast recombination time of the NCs, 1–2 ns (or even less under ASE conditions) [28], strongly limits the generation of *I*_ph_ before hopping transport can occur. Nevertheless, repeated reabsorption and re‐emission events effectively sustain the excitation over millimeter‐scale distances, which can be interpreted as an increase in the effective excitation persistence time during propagation [19]. Above the ASE threshold, the reabsorption of the B1 photons sustains the population required for optical gain, leading to amplification of the B2 emission band, which becomes the dominant long‐range transport channel.

The dependence of *I*_ph_ as a function of *z* is reproduced with the rate generation/equation model, considering that the measured *I*_ph_ is dominated by carriers generated through ASE, see Figure 3f. Particularly, the best fitting is obtained with mobility per pulse of 0.0027 cm^2^·V^−1^·s^−1^, close to that found with the modulated laser, *α*_1_ = 15 cm^−1^ and *α*_2_ = 8 cm^−1^, and parameters in Supporting Information S1: Table S5. The different absorption coefficients found in the case of the pulsed excitation are explained by the generation of optical gain. At the maximum peak power, the simulation indicates that the reabsorption of the B1 photons produces an optical gain *g* > 10 cm^−1^ along more than 1 mm [28]. This gain enables the propagation of an intense ASE photon flux over several millimeters (Supporting Information S1: Figure S11), thereby sustaining the nonlocal photocurrent experimentally observed in Figure 3f and reproduced by the simulations in Figure 3g.

## 5 Conclusions

In conclusion, this work demonstrates an effective strategy to enhance the photocurrent generation in MHP‐based PDs through PR processes. The device is based on a nanocomposite thin film of Ni(AcO)_2_ embedding distinct populations of MAPbBr_3_ NCs, which exhibits a strong room‐temperature PL and a measurable photocurrent under moderate excitation, while maintaining a dark current below 1 nA. The significant spectral overlap between the PL and absorption bands enables light reabsorption from smaller to larger NCs, resulting in a measurable flux of re‐emitted photons efficiently collected by the electrodes. Under modulated laser excitation (100 Hz–1 kHz), photon recycling enhances the photocurrent by approximately 60% through the redistribution of excitation along the nanocomposite waveguide. Under nanosecond pulsed excitation, the emergence of amplified spontaneous emission signal further boosts the photon‐mediated transport, resulting on a photocurrent of 0.8 mA per pulse. These findings highlight PR as a promising route to boost the efficiency of MHP photodetectors and to advance their integration into color and multispectral imaging technologies.

### 5.1 Experimental

Fabrication: MAPbBr_3_−Ni(AcO)_2_ thin films were synthesized by a single‐step procedure detailed elsewhere [29, 30]. Nickel acetate tetrahydrate (Ni(CH_3_COO)_2_·4H_2_O, 98% Alfa Aesar) was used as a precursor of the matrix, methylammonium bromide (CH_3_NH_3_Br, 99.5% Ossila) and lead (II) bromide (PbBr_2_, 98.0% Sigma‐Aldrich) as precursors of the perovskite NCs, and DMF (N, N‐Dimethylformamide anhydrous, 99.8% Sigma‐Aldrich) as a solvent. The typical Ni(AcO)_2_ precursor formulation consisted of a solution of 2 M Ni(AcO)_2_ in DMF (10 mmol in 5 mL of DMF). The precursor solution was aged in a dry bath block for 10 min at 70°C. The MAPbBr_3_ precursor was prepared by adding 5 mmol MABr and 5 mmol PbBr_2_ in 5 mL DMF. The desired concentrations were achieved by carefully mixing the solutions of Ni(AcO)_2_ and MAPbBr_3_ in a specific ratios incorporating DMF when necessary. The substrates (glass, Si, SiO_2_/Si) were rinsed with deionized water and followed by a cleaning step in a ultrasonic bath using a 3:1 solution consisting of isopropanol (99.5%, VWR Chemicals) and acetone (99%, VRW Chemicals) for 10 min. Prior deposition, substrates were dried with compressed air and subjected to ozone treatment for 10 min in a UV chamber to remove organic residues and prepare the surface. The in situ synthesis of the nanocomposite was carried out by spin‐coating the precursor solution onto the desired substrates at 3000 RPM for 40 s in a dry box (< 15% RH). Following the deposition, the films were then placed in a humidity chamber at 25°C and 52% RH for 15 min. During this process, the nanocomposite film exhibited a visible change in absorption (turning yellow) and strong photoluminescence (under a UV lamp) consistent with the formation of MHP NCs. The concentration of NCs in the matrix was controlled by adjusting the concentration of MAPbBr_3_ precursors.

The nanocomposite thin films were deposited on a commercial glass substrate (S161‐20, Ossila) with prepatterned ITO electrodes. Each substrate contains five sets of three interdigital electrodes with a fixed gap of 50 μm connected to 2 mm paths, see Figure 1c. After deposition, the part of the nanocomposite covering the paths was carefully cleaned to enable subsequent wire bonding.

Characterization: The photocurrent was measured under ambient conditions at room temperature placing the sample on an Ossila test board (P2008A1) connected to the appropriate system. The I–V curves were characterized by illuminating the samples with a continuous wave (CW) 450 nm laser with intensity ranged between 40 nW and 3 mW with neutral density filters and recording the photogenerated current as a function of the bias voltage using a Keithley Series 2400 Source Meter Unit (Keithley Instruments Inc.).

To characterize the frequency response, the samples were illuminated with a laser at 450 nm (MDL‐XS‐450, CNI laser), whose amplitude is externally modulated from 100 Hz to 2 MHz by a signal generator (TTI, model TPG3151). The transimpedance amplifier described elsewhere was used to convert *I*_ph_ in a voltage to be measured by an external SR810 DSP lock‐in amplifier (Stanford Research Systems).

The PL was measured by pumping the films with a modulated laser (100 Hz–1 kHz) at 450 nm or a Nd: YAG laser tripled to 355 nm (< 1‐ns pulses at 1‐kHz repetition rate) and collecting the forward‐scattered PL into a fiber optics connected to a commercial HR4000 Ocean Optics spectrograph. During the PL characterization as a function of the excitation power or propagation distance, *I*_ph_ was also characterized using a Keithley Series 2400 Source Meter Unit (Keithley Instruments Inc.).

## Author Contributions

**Sandra Soriano‐Diaz:** conceptualization, investigation, methodology, validation, software, formal analysis, data curation. **Jaume Noguera‐Gomez:** investigation, methodology. **Diego Ramirez‐Munoz:** investigation, visualization, writing – review and editing. **Pablo P. Boix:** project administration, resources. **Juan P. Martinez‐Pastor:** writing – review and editing, investigation, resources, project administration. **Rafael Abargues:** resources, investigation, validation, writing – review and editing. **Isaac Suarez:** conceptualization, investigation, funding acquisition, writing – original draft, writing – review and editing, visualization, validation, methodology, software, formal analysis, project administration, data curation, supervision, resources.

## Supporting information


Supporting Information S1

## Data Availability

The data that support the findings of this study are available from the corresponding author upon reasonable request.
