# Supplementary material for: Photon Recycling–Driven Photodetection Enhancement in Ni(AcO)2–MAPbBr3 Nanocomposite Waveguides
Source: Nanophotonics. 2026 Jul 2;15(13):e70196. doi: 10.1002/nap2.70196 (PMC13642050; doi:10.1002/nap2.70196)
Supplement: Supplementary file 1 [file NAP2-15-e70196-s001.pdf]

Photon Recycling–Driven Photodetection Enhancement in $Ni(AcO)_2$–$MAPbBr_3$ Nanocomposite Waveguides

Sandra Soriano-Díaz, [1,2] Jaume Noguera-Gómez, [2,3] Diego Ramírez-Muñoz, [1] Pablo P. Boix, [4] Juan P. Martínez-Pastor, [2] Rafael Abargues, [2] and Isaac Suárez [1,2*]

[1] *Departamento de Ingeniería Electrónica, Escuela Técnica Superior de Ingeniería, Universidad de Valencia, Valencia 46100, Spain*

[2] *UMDO, Instituto de Ciencia de los Materiales, Universidad de Valencia, Valencia 46980, Spain*
*e-mail: isaac.suarez@uv.es,
[3] *Energy Research Institute @ NTU, Nanyang Technological University, Research Techno Plaza, 637553, Singapore*
[4] *Instituto de Tecnología Química (Universitat Politècnica de València- Agencia Estatal Consejo Superior de Investigaciones Científicas)*

S1. Stability.

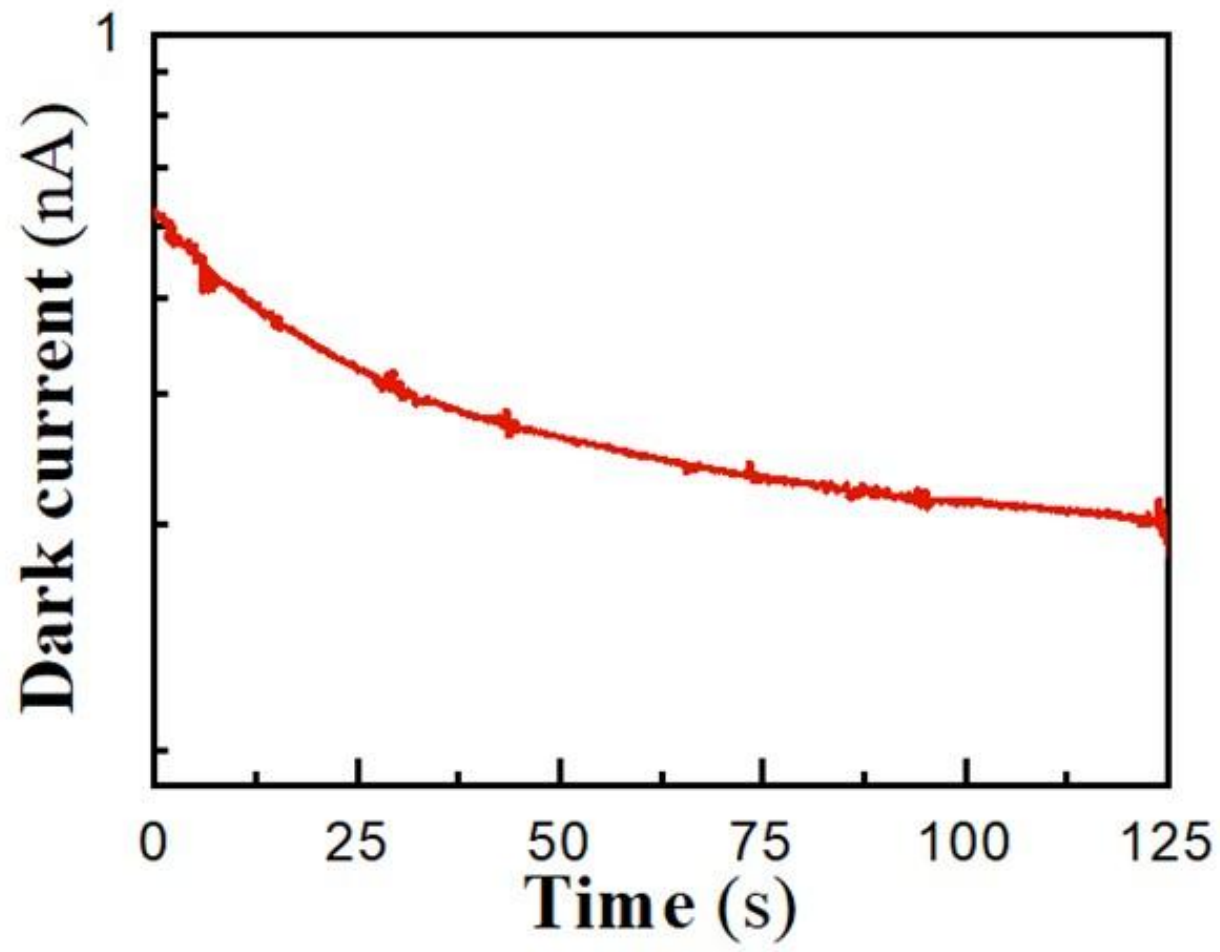

Figure S1. Dark current as a function of time.

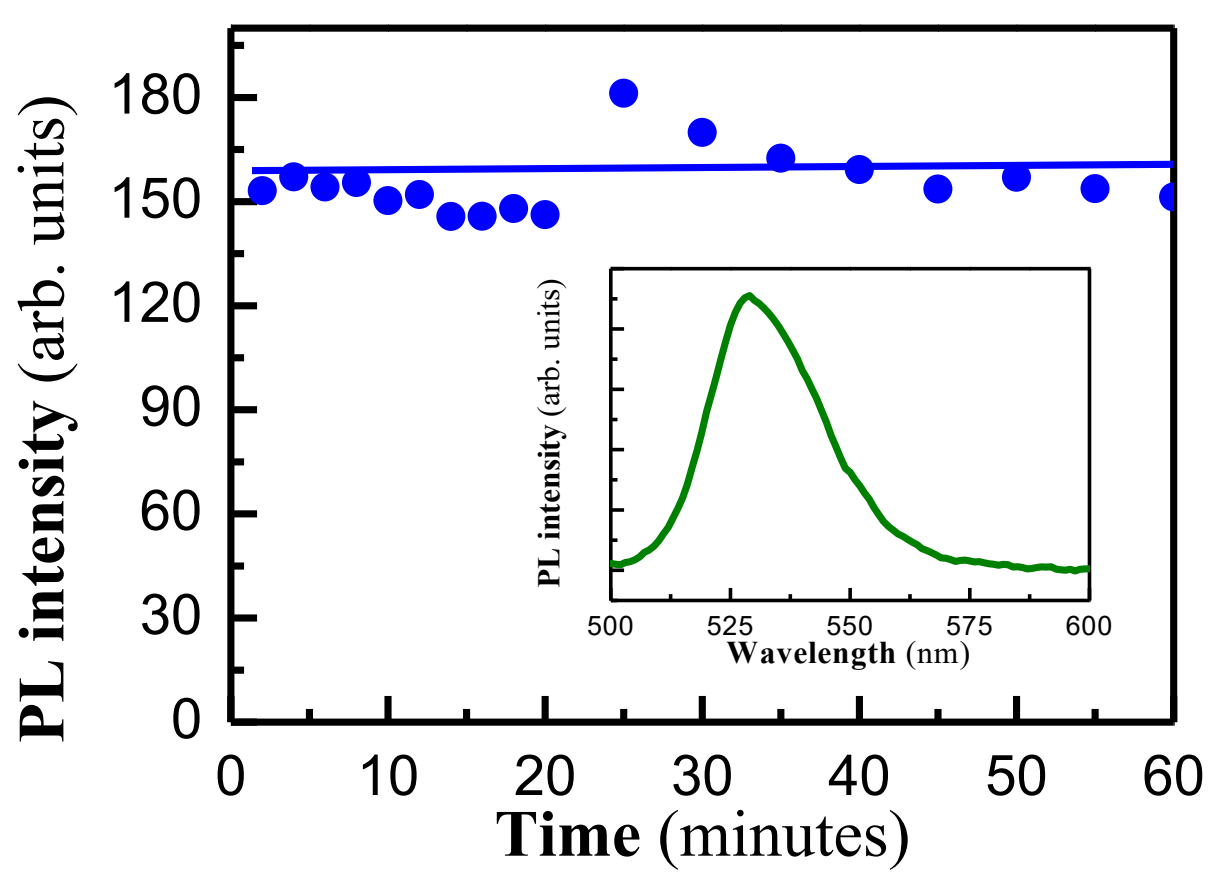

Figure S2. PL as a function of time under CW excitation.

S2. Mode Analysis.

Table S1 indicates the refractive indexes of the materials composing the photodetector used in this work. The refractive index of the nanocomposites was obtained following the approximation detailed elsewhere, while the refractive indexes of the ITO and glass were obtained from Palik. These values for the refractive index were used to calculate the effective refractive indexes of the modes in the waveguide considering thicknesses of 400 nm and 100 nm for the nanocomposite and ITO, respectively. Table S2 summarizes the TE0 mode in the air/nanocomposite/glass and air/nanocomposite/ITO/glass calculated for a nanocomposite with the optimal concentration of ff=0.4.

Table S1. Refractive indexes of the materials

Material	530 nm	545 nm
$Ni(AcO)_2$-$MAPbBr_3$	1.82652-0.01657i	1.7826-1e-5i
ITO	1.8833- 0.0034i	1.8685-0.0033i
Glass	1.52	1.52
Air	1	1

Table S2. Effective refractive indexes of the modes of nanocomposite waveguides with ff=0.4.

Material	Mode	530 nm	545 nm
Air/Nanocomposite/Glass	TE0	1.76011- 0.016i	1.71439 -0.000006i
	TM0	1.74184-0.016i	1.69614-0.000006i
Air/Nanocomposite/ITO/Glass	TE0	1.78726-0.014i	1.74769-0.0007i
	TM0	1.77585-0.014i	1.73513-0.0005i

S3. TEM.

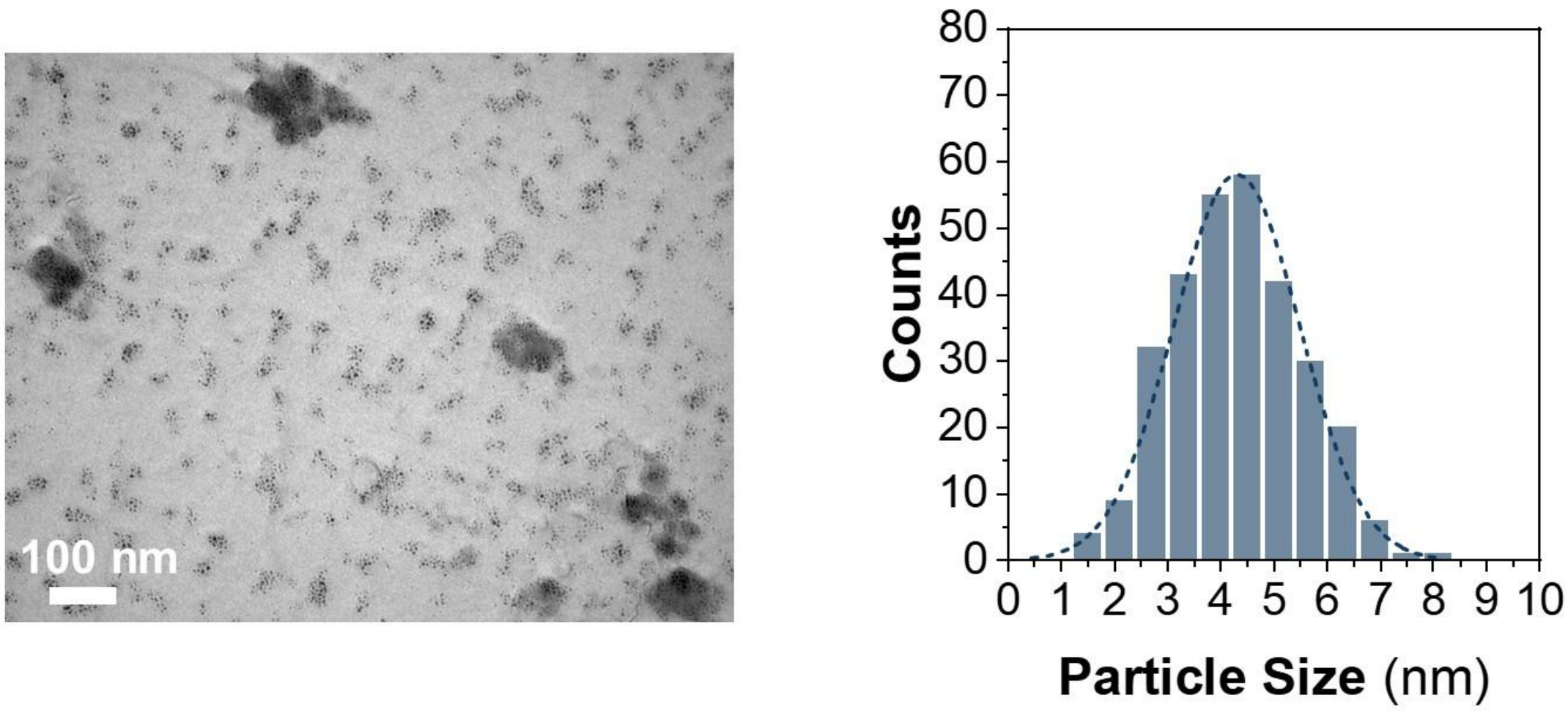

Figure S3. TEM image and statistics on particle size.

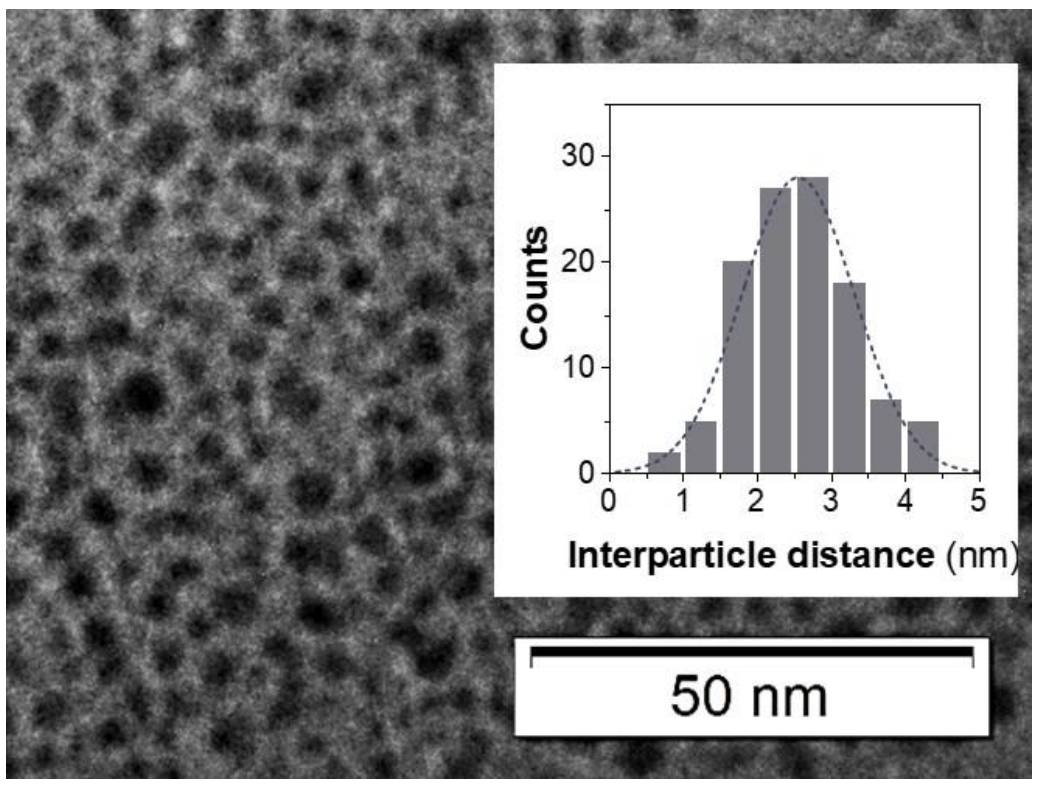

Figure S4. Zoom of TEM image and statistics on interparticle distance.

S4. Characterization of the electrical response.

The electrical response of the device under study is reproduced by the following transfer function representing a resistance R_0 in series with a R_1-C_1 parallel network, with a cut-off frequency given by $f_c=1/2\pi R_1 C_1$. See parameters of the modelling in Table S3.

$$z = R_0 + \frac{R_1}{1+\left(\frac{f}{f_C}\right)^N} \quad (1)$$

where the exponent N is included to account the non-ideal electrical behavior of the components.

Table S3. Model parameters of Z vs frequency.

	C=0.5 M	C=0.3 M
R_1	115 MΩ	900 MΩ
C_1	1.4 nF	0.2 nF
N	0.9	0.9
R_0	0.9 MΩ	1.1 MΩ

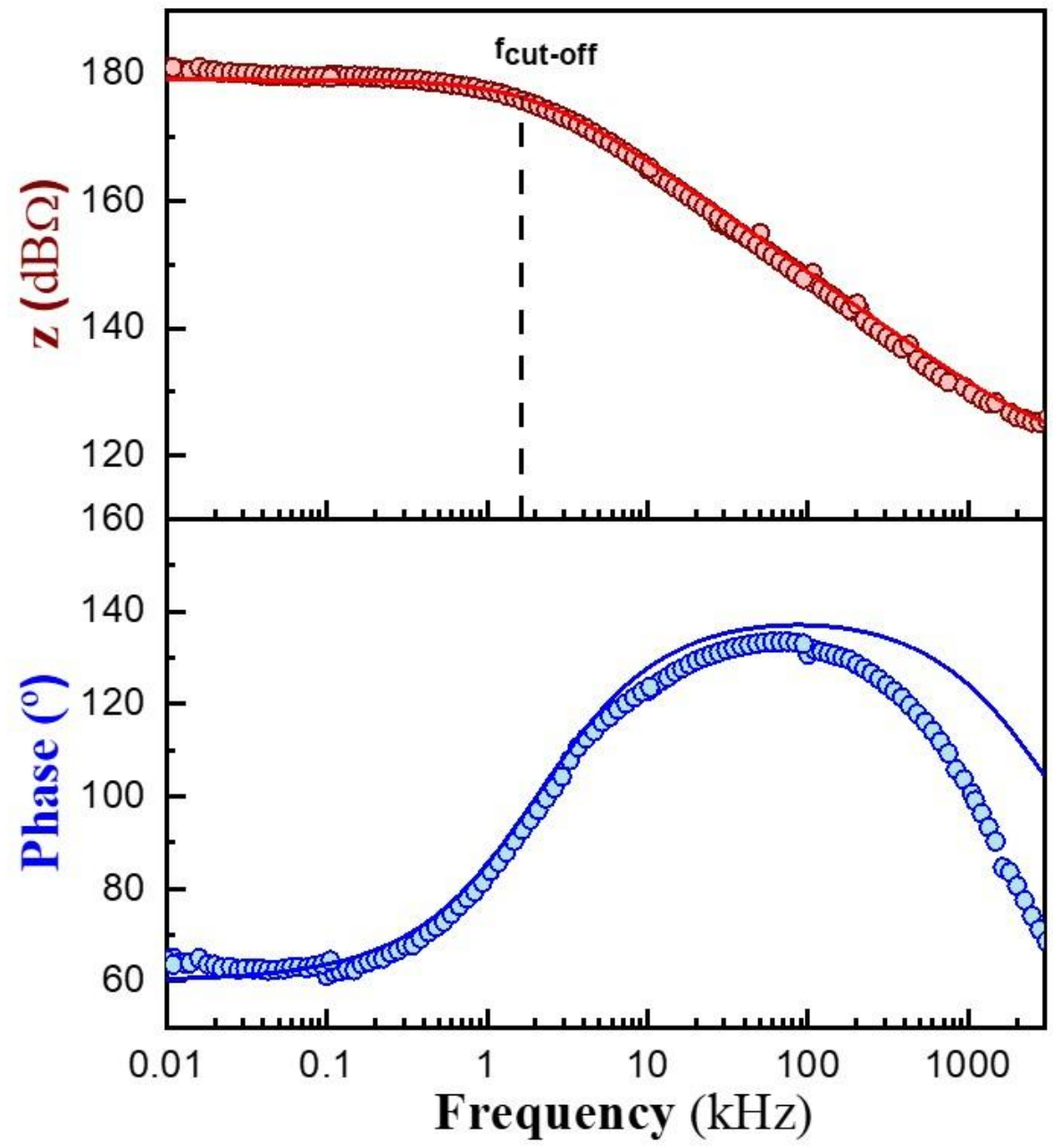

Figure S5. Analysis of the impedance in a nanocomposite with C=0.3 M.

S5. Characterization of the I-V curves.

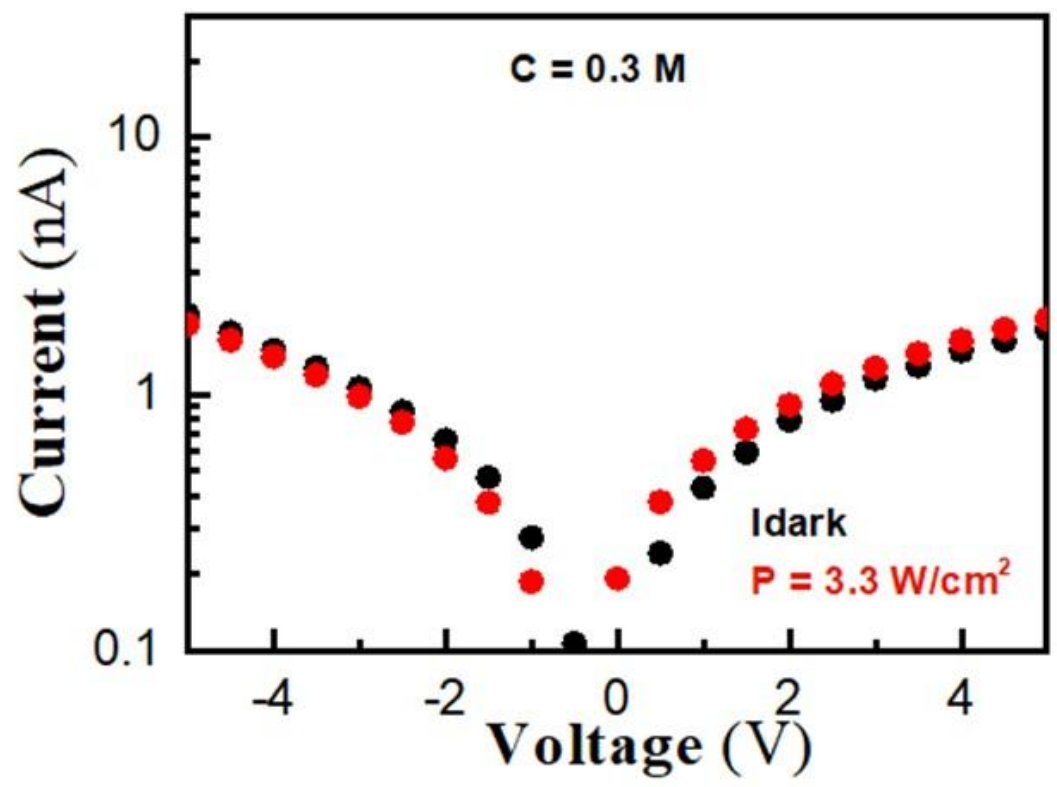

Figure S6. I-V curve of photodetector with C=0.3 M.

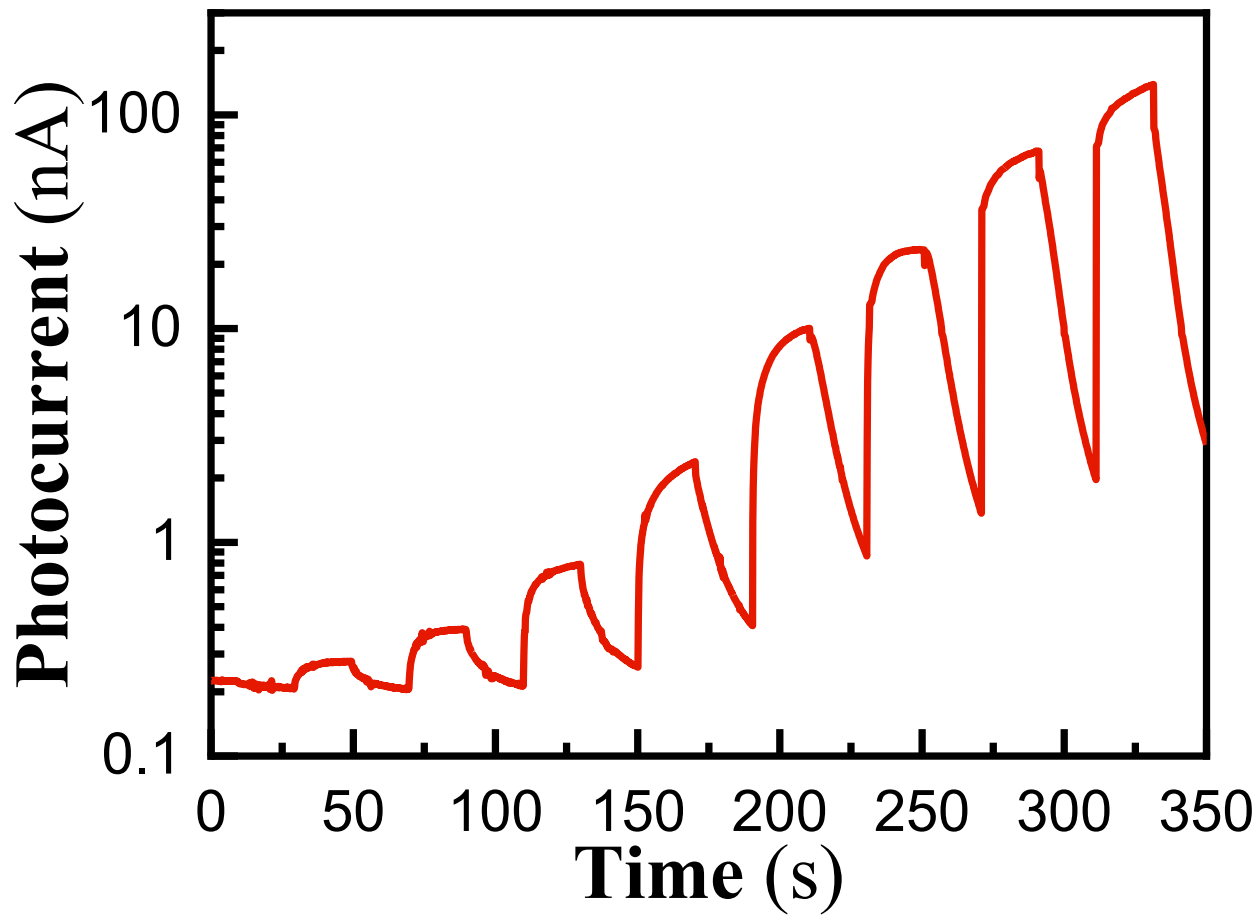

Figure S7. ON-OFF I_{ph}.

S6. Characterization of the frequency response.

The experimental amplitude/phase of I_{ph} as a function of the frequency was reproduced by a transfer function composed by three fractional poles:

$$\frac{i_{ph}}{i_{ph0}} = \frac{1}{\left(1+\left(j\frac{w}{w_{c1}}\right)^{N1}\right)\cdot\left(1+\left(j\frac{w}{w_{c2}}\right)^{N2}\right)\cdot\left(1+\left(j\frac{w}{w_{c3}}\right)^{N3}\right)} \quad (2)$$

where i_{ph0} is the photocurrent at DC and ω_{ci} and N_i (i=1,2,3) are fitting parameters. Each fractional frequency is related with a RC network by ω_{ci}=//R_iC_i), representing the behaviour of the photodetector device (R_1, C_1, R_2 and C_2) and the transimpedance amplifier (R_3 and C_3) used in the measurements. Table S4 indicates the fitting parameters.

Table S4. Model parameters of i_{ph} vs frequency.

R1	50 MΩ	R2	0.1 Ω	R3	43 kΩ
C1	80 pF	C2	0.1 pF	C3	4.3 pF
N1	0.68	N2	0.5	N3	1

S6. Modelling.

The generation of the exciton density (N) at each propagation step is calculated together with the propagation of the primary (S_1) and secondary (S_2) flows of photons by using the following rate equation:

$$\frac{\partial N(z)}{\partial t} = G(z) - (A_{nr} + A_{r1} + A_{r2}) \cdot N(z) - g \cdot \frac{S_2(z)}{h\nu_2} + H(z) \quad (3)$$

where A_r is the radiative recombination rate (inverse of the recombination time), A_{nr} is the nonradiative recombination rate, S the photon density, $h\nu$ the photon energy, and the subindex i refers to the parameters of the first (i=1) and second (i=2) family of photons at 530 and 545 nm, respectively, and g the optical gain of the secondary flux of photons given by:

$$\mathrm{g(z)} = \Gamma \cdot \sigma \cdot (N(z) - N_0) \quad (4)$$

where Γ is the confinement factor (around 0.9 according to the mode distribution), N_0 the carrier density and σ the emission cross section. In the equations listed above, $A=A_{nr}+A_{r1}+A_{r2}=0.6$ are fixed to 0.56 following the kinetics analysis developed in the reference 30 of the main manuscript, where a lifetime of ≈1 ns was obtained for this filling factor; A_{nr} is set as 0.1 to consider a quantum yield of 80 %.

The model contemplates the following mechanisms to feed the exciton density:

$$G(z) = \frac{P(z)}{h\nu_p} \cdot \alpha_p \quad (5)$$

$$H(z) = \frac{S_1(z)}{h\nu_1} \cdot \alpha_1 \quad (6)$$

$$R(z) = \frac{S_2(z)}{h\nu_2} \cdot \Gamma \cdot \sigma \cdot N_0 \quad (7)$$

Here, G(z), H(z) and R(z) correspond to the standard absorption of the pump fluence at 355 nm, the recycling of the first flow of photons at 530 nm, and the reabsorption of the ASE signal at 545 nm, respectively.

- The first excitation mechanism is modeled with a Gaussian distribution, $P(z)=P_0 \cdot \exp(-2 \cdot (z-z_0)^2/w_0^2)$, where P_0 is the power density, z_0 the position of the spot on the sample and w_0 the width of the spot on the surface of the sample.
- The second mechanism depends on the propagation of the flux of PL photons along the structure, given by:

$$\frac{dS_1(z)}{dz} = -\alpha_2 \cdot S_1(z) + A_{r1} \cdot \frac{\Omega_1}{4 \cdot \pi} \cdot N(z) \cdot h\nu_1 \quad (7)$$

- Finally, the third mechanism is included in the gain equation (4), and contemplates the propagation of the second flux of photons, S_2, that gives rise to ASE generation.

$$\frac{dS_2(z)}{dz} = (\Gamma \cdot \sigma \cdot (N(z) - N_0) - \alpha_2) \cdot S_2(z) + +A_{r2} \cdot \frac{\Omega_2}{4 \cdot \pi} \cdot N(z) \cdot h\nu_2 \quad (8)$$

In the equations listed above, Ω/4π is spontaneous emission factor, the parameters α and hν are to the absorption losses and photon energy, respectively; and the subindex p, 1 and

2 refer to the pump beam at 355 nm, the PL photons at 530 nm and the PL photons at 545 nm, respectively.

Equation (3) is solved in the stationary state together with equations (7-8) to obtain the distribution of the carriers, N(z), and photons, $S_1(z)$ and $S_2(z)$, for a given position of the excitation spot (z_0). At these conditions, the number of carriers can be written as:

$$N(z, z_0) = \frac{G(z,z_0)+H(z,z_0)+R(z,z_0)}{A+\Gamma\cdot\sigma\cdot\frac{S_2(z,z_0)}{h\nu_2}} \quad (9)$$

where $N(z,z_0)$ can be decomposed into three contributions: $N_G(z,z_0)$, $N_H(z,z_0)$ and $N_R(z,z_0)$, corresponding to the direct illumination, reabsorption of the spontaneously emitted photons and reabsorption of the ASE photons:

$$N_G(z, z_0) = \frac{G(z,z_0)}{A+\Gamma\cdot\sigma\cdot\frac{S_2(z,z_0)}{h\nu_2}} \quad (10)$$

$$N_H(z, z_0) = \frac{H(z,z_0)}{A+\Gamma\cdot\sigma\cdot\frac{S_2(z,z_0)}{h\nu_2}} \quad (11)$$

$$N_R(z, z_0) = \frac{G_R(z,z_0)}{A+\Gamma\cdot\sigma\cdot\frac{S_2(z,z_0)}{h\nu_2}} \quad (12)$$

Here, since the hopping time between nanocrystals limits the electrical conductivity of the nanocomposite, each contribution is considered as a transport channel that contributes differently to the measured photocurrent, I_{ph}. In particular, the interplay between electronic hopping, carrier lifetime and the propagation of photons determines the relative efficiency with which each contribution is converted into collected current. In this way, we define an effective carrier density, $N_{eff}(z_0)$, as the carrier population effectively contributing to the photocurrent within the electrode region:

$$N_{eff}(z_0) = \frac{1}{L}\int_{electrode}(k_G \cdot N_G(z, z_0) + k_H \cdot N_H(z, z_0) + k_R \cdot N_R(z, z_0))dz \quad (13)$$

where the coefficients k_G, k_H and k_r (ranging from 0 to 1) account for the transport efficiency of each channel in overcoming hopping-limited transport and contributing to charge collection. The integration is performed over the electrode length (L) for a given position of the excitation spot (z_0)

For a given carrier distribution, $N_{eff}(z_0)$, the photocurrent, $I_{ph}(z_0)$, is calculated as:

$$\frac{I_{ph}(z_0)}{A} = e \cdot \mu_n \cdot N_{eff}(z_0) \cdot \frac{V}{gap} \quad (14)$$

where e is the electron charge, μ_n the electron mobility, A the area of the electrodes, V the applied voltage and *gap* the gap between the electrodes. Finally, carrier diffusion is

neglected because the length of the waveguide, ≈1 mm, is much longer than the diffusion length of carriers in the perovskite.

The aforementioned model allows us to fit the experimental data under modulated and pulsed excitation. Table S5 summarizes the fitting parameters.

Table S5. Model parameters.

	450 nm (modulated)	355 nm (1 ns pulsed)
$A_{r1}+A_{r2}$	0.5	0.5
A_{nr}	0.1	0.1
$A_{r1}\cdot\Omega_1$	0.4975	0.4975
$A_{r2}\cdot\Omega_2$	$5\cdot10^{-3}$	$5\cdot10^{-4}$
Γ	0.9	0.9
σ_e (cm^{-2})	0	$1.1\cdot10^{-19}$
N_0 (cm^{-3})	$1.5\cdot10^{17}$	$1.5\cdot10^{17}$
α_p (cm^{-1})	$2\cdot10^4$	$2\cdot10^4$
α_1 (cm^{-1})	10	27
α_2 (cm^{-1})	1	9.5
k_G	0.22	0
k_H	1	0
k_R	0	1
μ ($cm^2\cdot V^{-1}\cdot s^{-1}$)	$7.8\cdot10^{-4}$	$2.2\cdot10^{-7}$ *
n_0 (cm^{-3})	$5.4\cdot10^{11}$	$1.9\cdot10^{15}$ *

*per pulse

S7. I_{ph} measurements under a modulated beam.

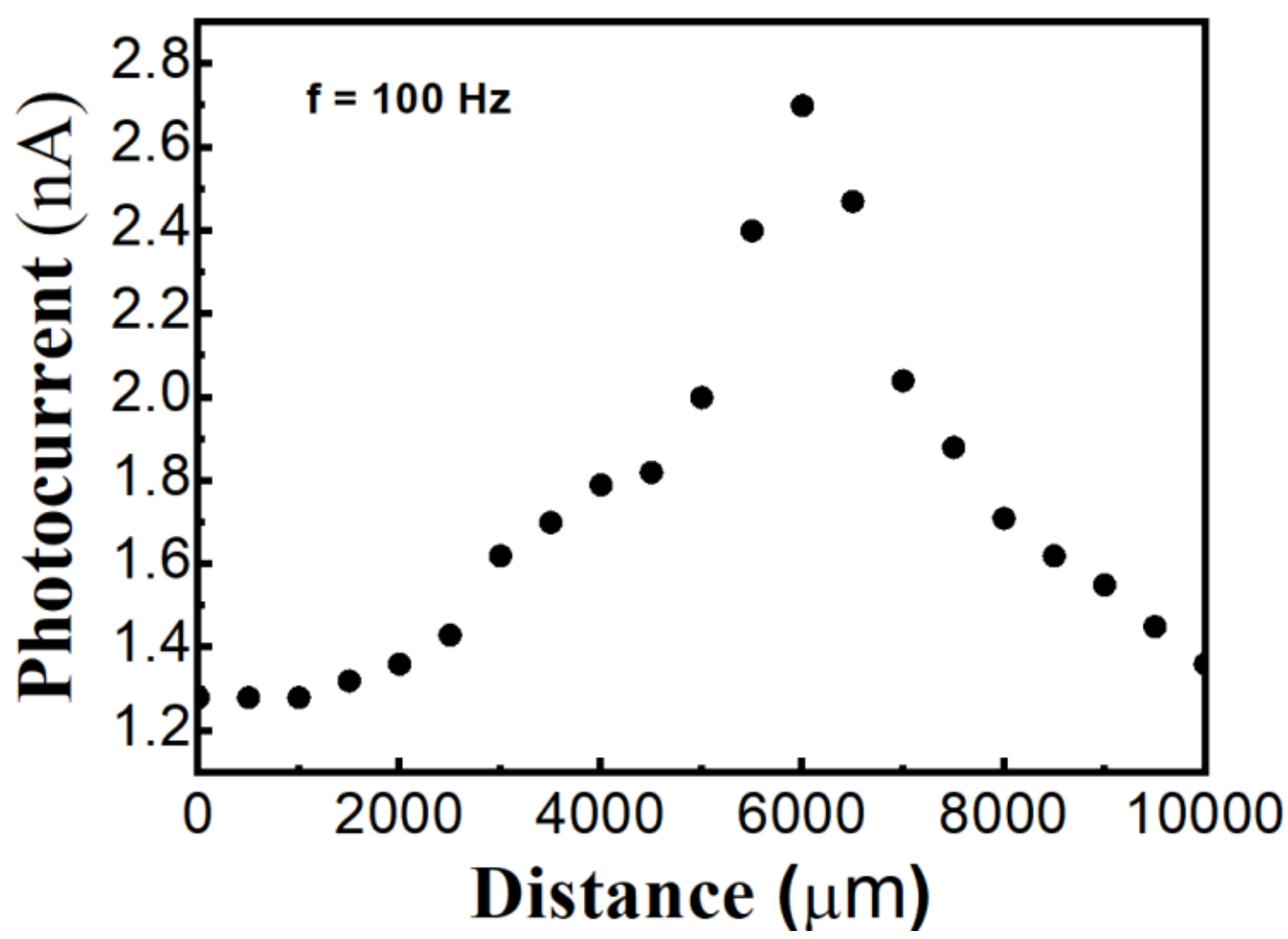

Figure S8. I_{ph} as a function of z excited with a modulated laser at 100 Hz with a duty cycle of 50 %.

S8. Influence of traps.

Equation (3) can include the influence of traps by adding a trap population at each propagation distance/time, n_t, a maximum trap density, N_t, and a trapping, τ_c, and detrapping, τ_e, times:

$$\frac{\partial N(z)}{\partial t} = G(z) - A_r \cdot N(z) - g \cdot \frac{S_2(z)}{h\nu_2} + H(z) + \frac{N(z)}{\tau_e} - \frac{N(z)}{N_t} \cdot \left(1 - \frac{n_t}{N_t}\right) \cdot \frac{1}{\tau_c} \quad (15)$$

$$\frac{dn_t}{dt} = -\frac{N(z)}{\tau_e} + \frac{N(z)}{N_t} \cdot \left(1 - \frac{n_t}{N_t}\right) \cdot \frac{1}{\tau_c} \quad (16)$$

Equations (15-16) are solved together with equations (7-10) with a Runge-Kutta algorithm to understand the influence of traps in the generation of I_{ph}. For this purpose, the simulation considers an initial excitation at t=0 and simulates the total I_{ph} with the number of carriers, N(z) averaged along the whole temporal window. This assumption corresponds to the realistic scenario depicted in Figure 3 of the main manuscript, in which the measurement is carried out until a stationary current is reached at the SMU. For the case of fast trapping and slow recovery ($\tau_e/\tau_c = 10^3$), the simulations confirm photon recycling as the dominant mechanism for the generated photocurrent (I_{ph}). For example, a moderate trap density ($N_t = 10^{11}$-10^{12} cm^{-3}; Figure S5a) reproduces the stationary behavior reported in the main manuscript, whereas only a substantial increase in trap density (Nt = 10^{14} cm^{-3}) leads to a suppression of I_{ph}. On the other hand, increasing τ_e results in only a minor reduction of I_{ph}, without significantly altering the overall behavior, as illustrated in Figure S5b.

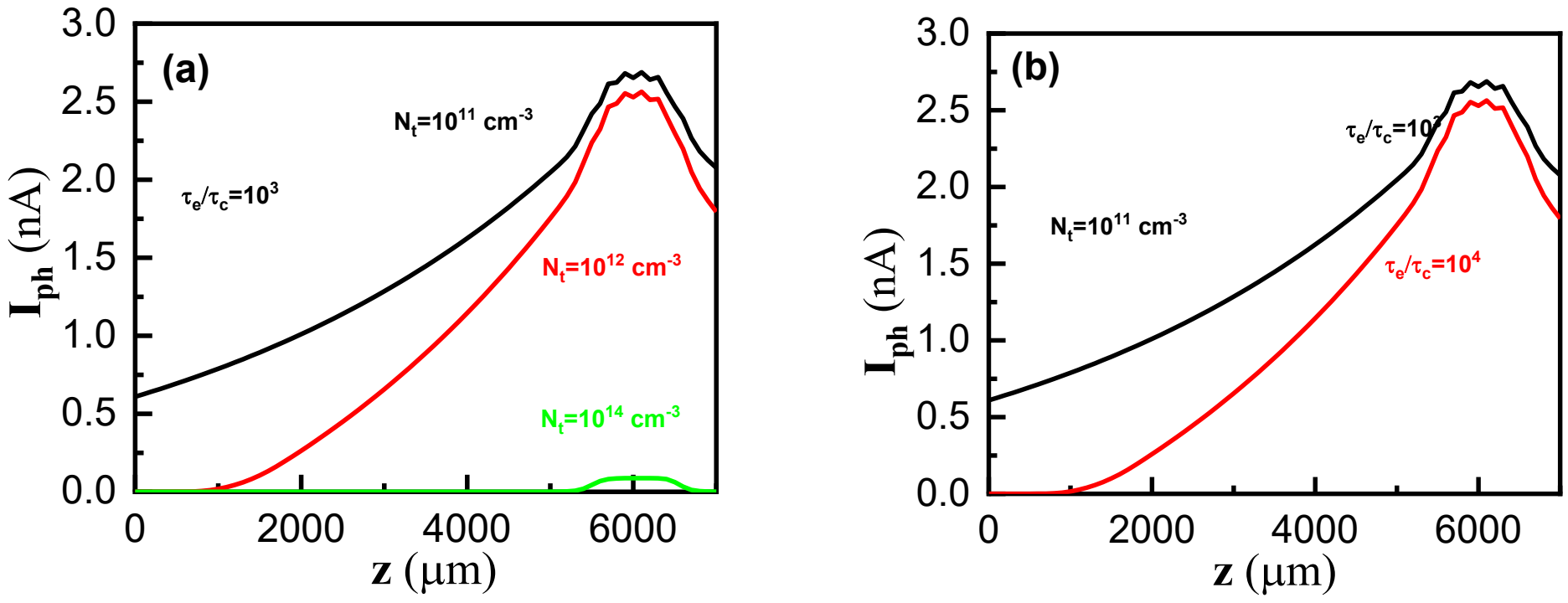

Figure S9. Simulation of I_{ph} with trap states.

S9. I_{ph} measurements under a pulsed beam.

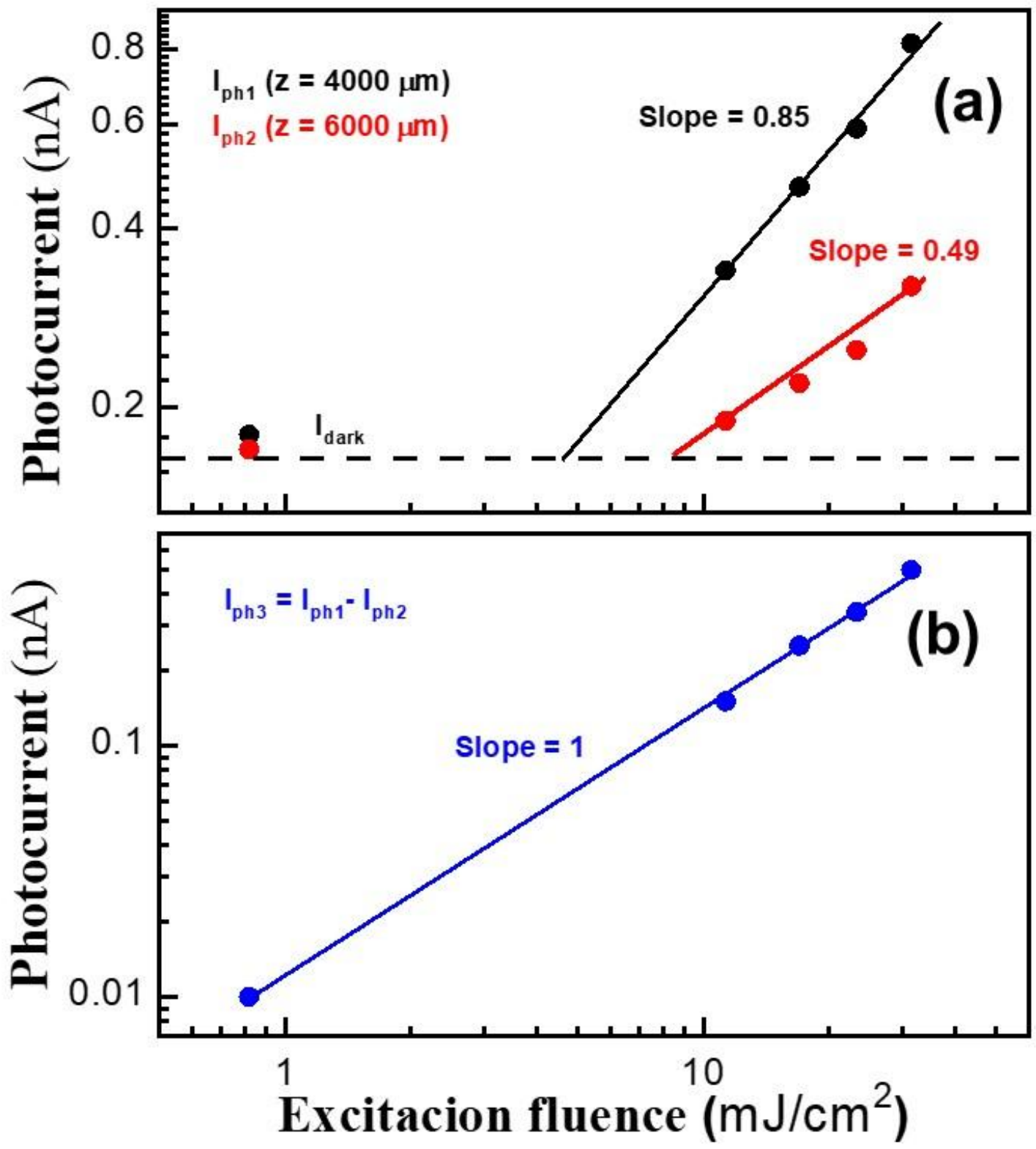

Figure S10. I_{ph} as a function of the excitation fluence

S10. Simulation of direct pump, PR photons and ASE along the waveguide.

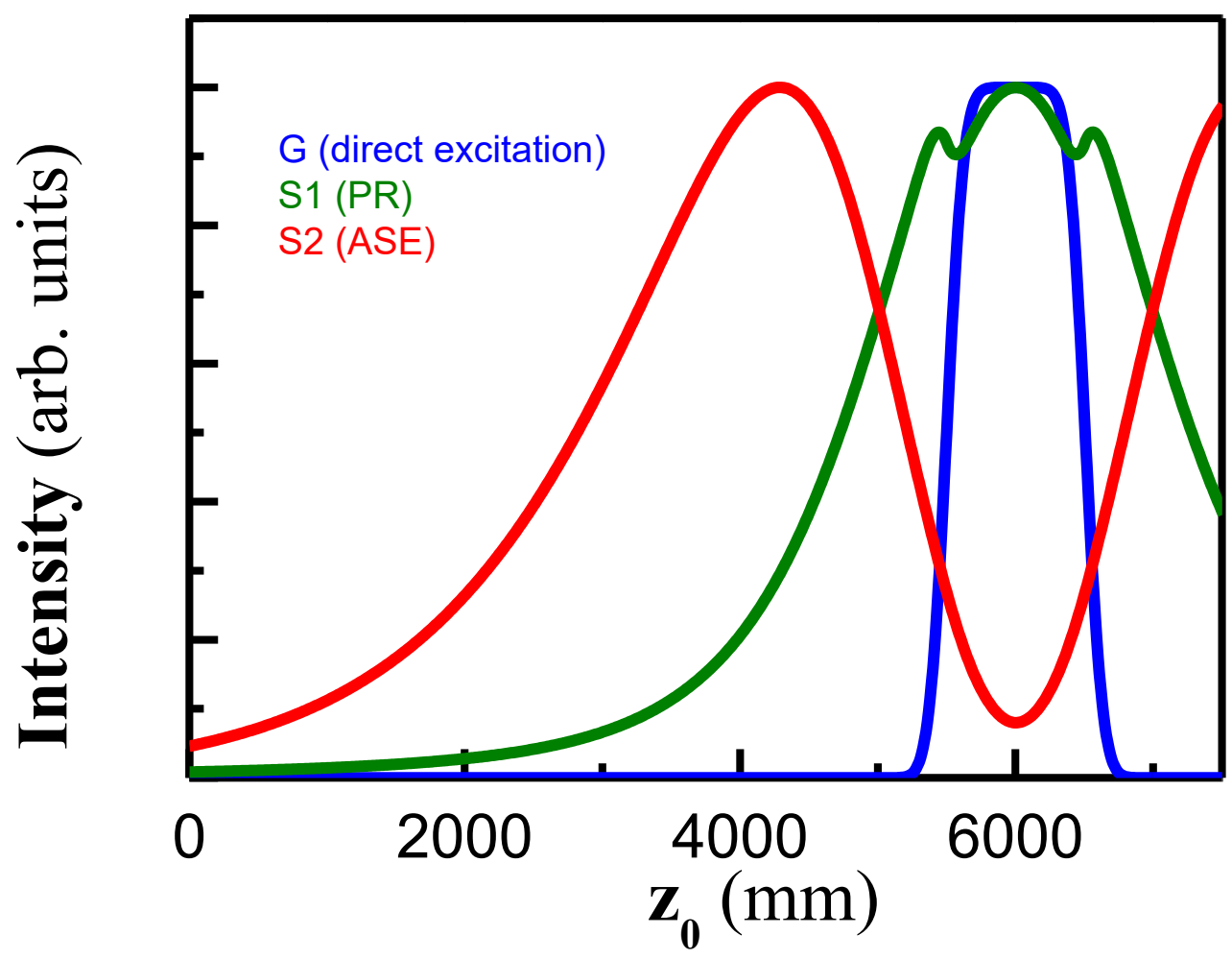

Figure S11. Normalized photon density of the direct excitation (blue), recycled photons (green) and ASE photons (red).
